# An Unusual Case of Nonbacterial Thrombotic Endocarditis Attributable to Malignancy

**DOI:** 10.7759/cureus.44734

**Published:** 2023-09-05

**Authors:** Luis E Santiago, Winy Kujundzic, Stephanie Wong, Samyukta Swaminath, Pallavi Aneja

**Affiliations:** 1 Internal Medicine, Hospital Corporation of America (HCA) Florida Westside Hospital, Plantation, USA; 2 Public Health, Emory University, Atlanta, USA; 3 Osteopathic Medicine, Nova Southeastern University, Davie, USA

**Keywords:** transesophageal echocardiogram, embolic infarction, embolic cva, non-bacterial thrombotic endocarditis, cardiac embolism, marantic endocarditis

## Abstract

Nonbacterial thrombotic endocarditis (NBTE), also known as marantic endocarditis, is a condition characterized by the deposition of thrombi and fibrin on normal or degenerated cardiac valves in the absence of microorganisms. We report a case of a 60-year-old male with nonbacterial thrombotic endocarditis found on transesophageal echocardiogram (TEE) after a normal TEE just one month prior. Our patient presented with abdominal pain associated with poor appetite and unintentional 20-pound weight loss for one month. Chest computed tomography revealed the presence of a mass-like opacification in the right lung middle lobe with moderate pericardial effusions. A biopsy of the mass confirmed malignancy consistent with lung primary adenocarcinoma. Subsequently, during hospitalization, the patient developed left lower extremity pain. Arterial ultrasound showed occlusion of the distal left popliteal artery for which he underwent thrombectomy of the left superficial femoral artery, balloon angioplasty of the left posterior tibial artery, and left popliteal artery. Repeat TEE during current hospitalization revealed a large 2 cm vegetation on the noncoronary cusp of the aortic valve. Studies for infective endocarditis were unremarkable. Subsequently, he was treated with aortic valve replacement and anticoagulation. After discharge, he returned with bilateral occipital infarcts four days later and expired.

## Introduction

Nonbacterial thrombotic endocarditis (NBTE), also known as marantic endocarditis, is a condition characterized by the deposition of thrombi and fibrin on normal or degenerated cardiac valves in the absence of microorganisms [1]. NBTE is commonly observed as a postmortem finding but can also present clinically, particularly in patients with underlying malignancies [2]. The reported incidence of NBTE ranges from 0.3% to 9.3% [3]. The incidence of visceral embolism varies from 14.1% to 90.9% [3].

The exact etiology of NBTE remains unclear, but several factors contribute to its development. Endothelial injury and hypercoagulability are believed to play synergistic roles in the pathogenesis of NBTE [2]. Endothelial injury can result from various conditions, including chronic inflammation, autoimmune diseases, and malignancies [4]. In the case of malignancy-associated NBTE, tumor cells release procoagulant factors that promote a hypercoagulable state [5].

Underlying malignancy is a significant risk factor for NBTE, with solid tumor cancers being commonly associated with its development [5]. Autopsy reports have indicated that patients with underlying malignancies are six times more likely to develop NBTE than the general population (1.25% vs. 0.2%) [4]. Among malignancies, mucin-secreting adenocarcinomas are particularly linked to NBTE [2]. The high association between malignancy and NBTE may be attributed to the release of procoagulant factors by tumor cells, promoting the formation of thrombi on cardiac valves [4].

Moreover, chronic inflammation plays a role in the development of NBTE. Conditions such as systemic lupus erythematosus and antiphospholipid syndrome have been associated with an increased risk of NBTE [6]. In these inflammatory states, circulating immune complexes and antiphospholipid antibodies can activate platelets and promote the formation of thrombi on cardiac valves [6].

The pathophysiology of NBTE involves the formation of sterile vegetations composed of agglutinated platelets mixed with strands of fibrin [4]. Unlike infective endocarditis, NBTE vegetations do not elicit an inflammatory reaction at the deposition site, making them prone to embolization and leading to systemic complications [4].

## Case presentation

**Figure 1:**
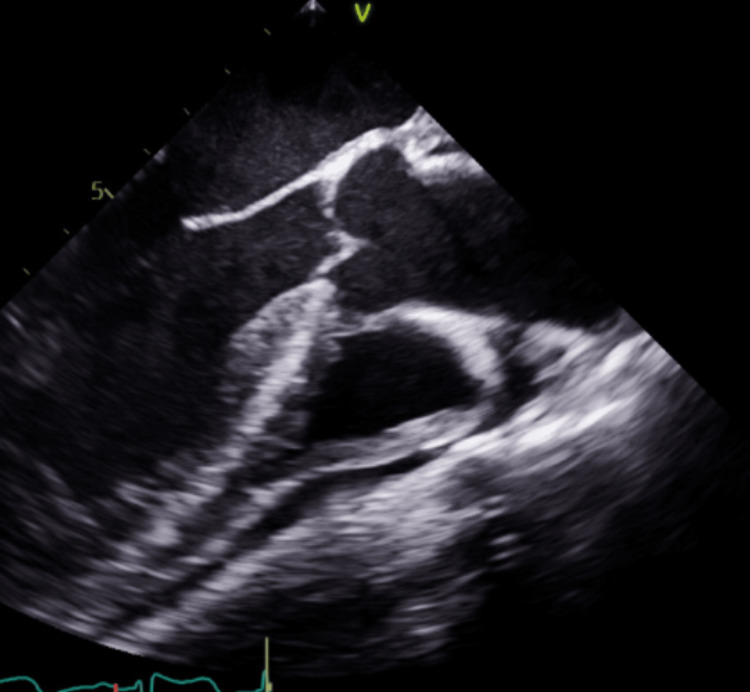
TEE performed 40 days before current TEE. Mid-esophageal long-axis view showing no vegetation on aortic valve. TEE: transesophageal echocardiogram

A 60-year-old male patient with a past medical history of tobacco smoking, alcoholism, substance abuse, cerebrovascular accident (CVA), and hypertension presented to our institution with abdominal pain with two days of evolution associated with poor appetite and a 20-pound weight loss in the last month. The patient described the pain as constant, diffuse, dull, 5 of 10 in intensity, and with no modifying factors. Forty days before this admission, he had been admitted and diagnosed with a CVA. TEE performed on that admission was unremarkable (Figure 1). He denied nausea, vomiting, diarrhea, fever, or any other complaints.

On physical examination, he had a good general appearance, was alert, awake, oriented, and was in no acute distress. There was tachycardia, normal heart sounds, and no murmur on cardiac examination. Lungs were clear to auscultation. On abdominal examination, there was diffuse tenderness to deep palpation, no distention, no mass/organomegaly, and no rebound. Pulses were present throughout all extremities. There were no other changes in other organ systems. Blood pressure was 166/72 mmHg, temperature 98.6°F, heart rate was 115 beats/min, respiratory rate was 18 breaths/min, and oxygen saturation was 98%.

Laboratory tests revealed hemoglobin of 11.1 g/dL, platelet count of 151,000/mm^3^, white blood cell count of 9,100/mm^3^ with 80% neutrophils, erythrocyte sedimentation rate (ESR) of 40 mm/h. Urinalysis revealed 11-20 RBC/hpf. All other blood work was unremarkable on admission.

**Figure 2:**
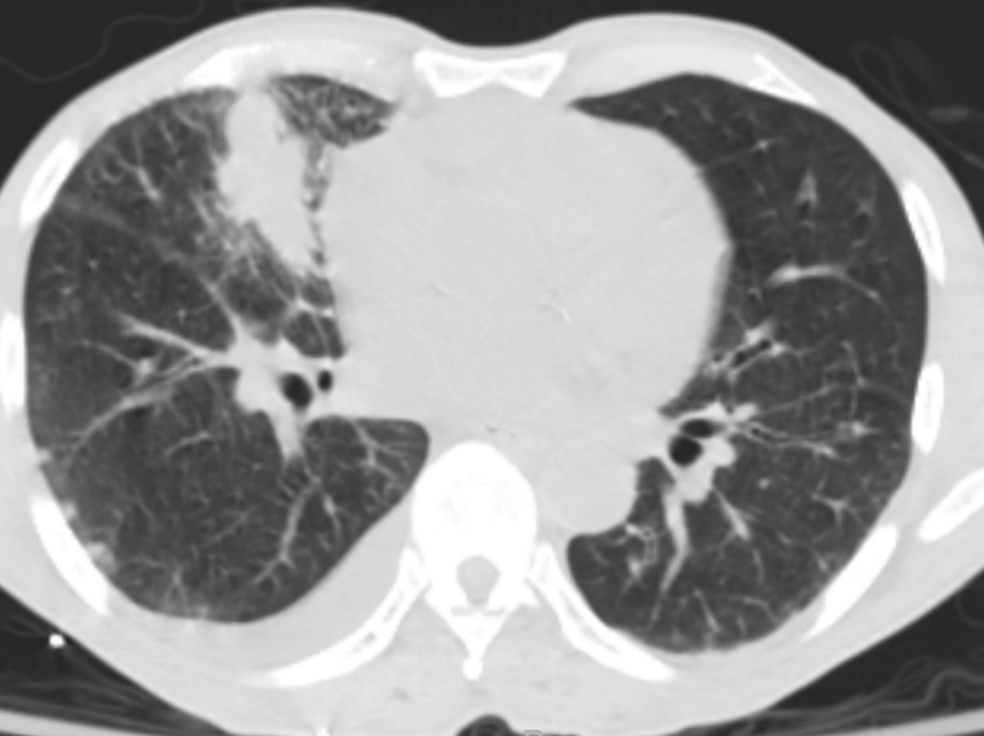
Chest computed tomography of the patient. CT scan of the whole chest showed a mass-like opacification in the right middle lobe, diffuse interlobular septal thickening throughout the right lung, and several nodular foci in the right lung.

A CT scan of the whole chest showed a mass-like opacification in the right middle lobe, diffuse interlobular septal thickening throughout the right lung, several nodular foci in the right lung, and moderate-sized pericardial effusion of simple fluid density (Figure 2).

Transthoracic Doppler echocardiography, using M-mode and two-dimensional imaging, revealed aortic valve with moderate regurgitation and a moderate pericardial effusion. No vegetation was noted on the aortic valve. There were no other abnormal findings visualized. After findings on echocardiography, the patient was started on prednisone and colchicine due to suspicion of pericarditis.

CT-guided biopsy of the right lung mass was performed and showed morphological and immunohistochemical findings were consistent with involvement by adenocarcinoma. Immunohistochemical stains performed showed the tumor cells staining positive for pan-keratin, cytokeratin (CAM 5.2), cytokeratin 7 (CK7), transcription termination factor 1 (TTF-1), and Napsin A.

**Figure 3:**
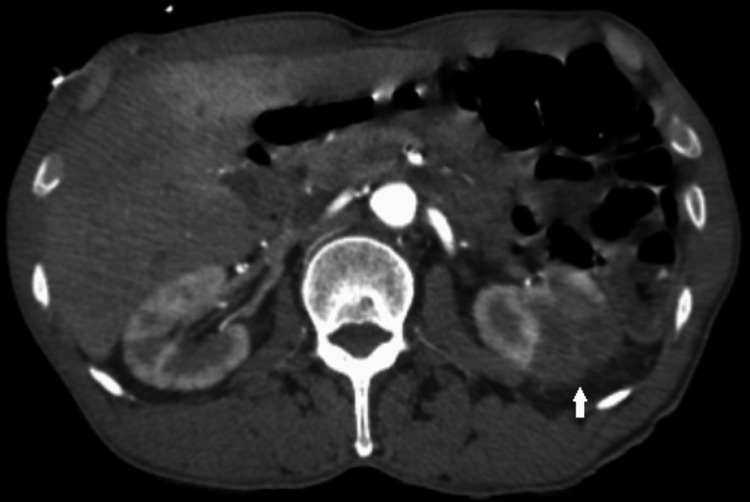
A CTA of the abdomen showed multiple wedge-shaped parenchymal hypodensities in kidneys (arrow). CTA: computed tomography angiography

**Figure 4:**
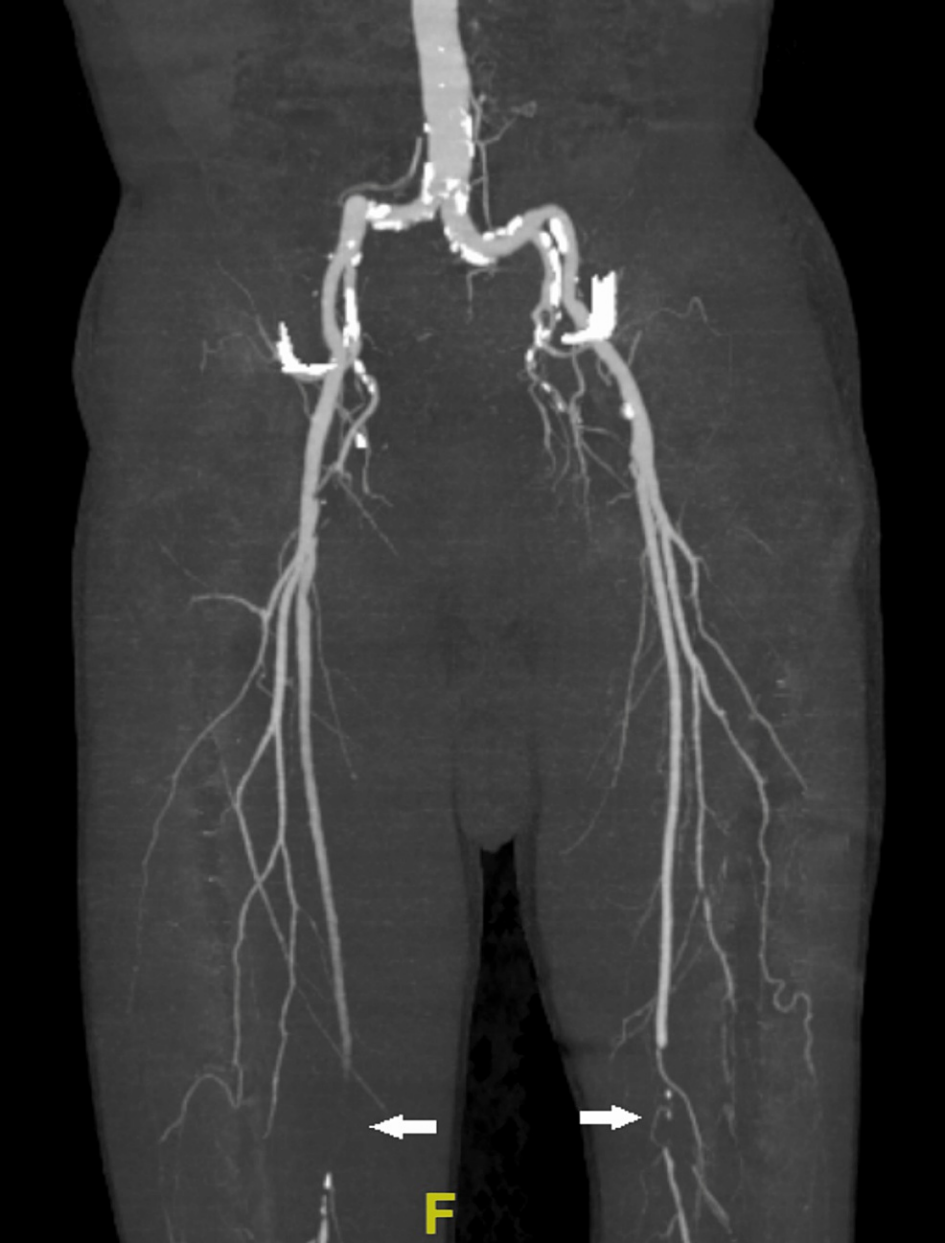
CTA of the abdomen with run-off showed complete occlusion of the right and left superficial femoral arteries (arrows). CTA: computed tomography angiography

As of the 11th day of admission, the patient complained of nonradiating, 5 of 10 in intensity, constant dull pain on the dorsal aspect of his left foot. Run-off abdominal computed tomography angiography (CTA) showed multiple wedge-shaped parenchymal hypodensities in the bilateral kidneys (Figure 3). There is complete occlusion observed in the right and left superficial femoral arteries, right and left posterior and anterior tibialis arteries, and left popliteal artery. All other arteries were patent (Figure 4).

After the findings on abdominal computed tomography, the patient was started on ceftriaxone and vancomycin, and two sets of blood cultures were taken. No growth was observed after five days of incubation. Extensive workup for blood culture-negative endocarditis was performed and was unremarkable.

The patient underwent primary percutaneous thrombectomy of the left superficial femoral artery, left tibioperoneal trunk, and left proximal posterior tibial artery, balloon angioplasty of the left posterior tibial artery and tibioperoneal trunk, and balloon angioplasty of the left popliteal artery.

**Figure 5:**
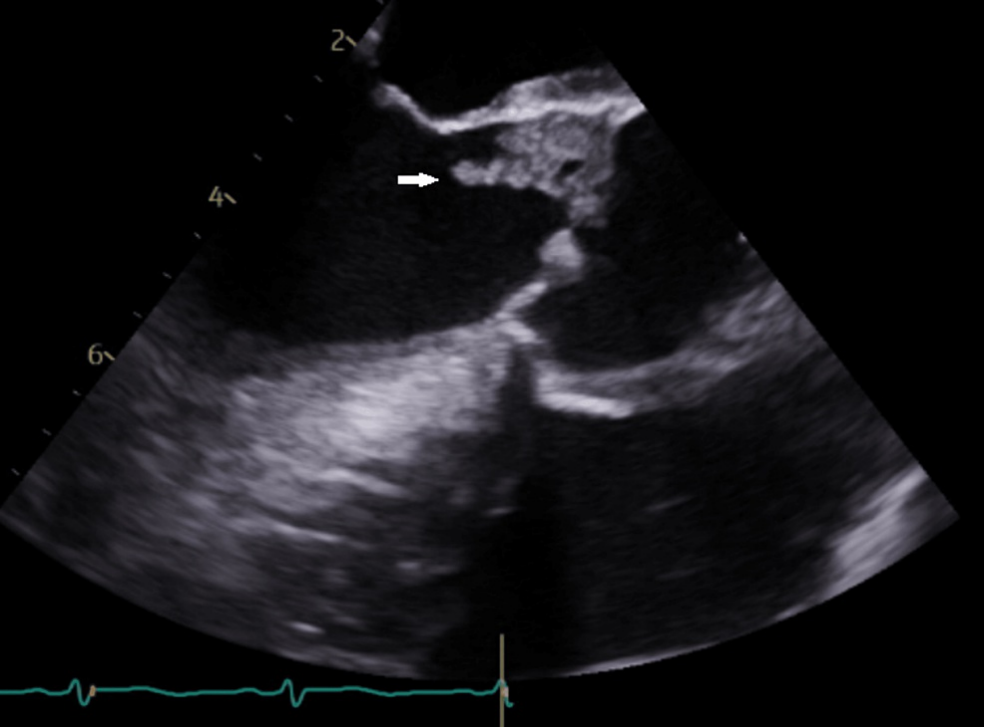
TEE showed the presence of a large vegetation. Large vegetation had both sessile as well as a mobile elongated piece measuring about 2 cm and was attached to the noncoronary cusp of the aortic valve (arrow). TEE: transesophageal echocardiogram

TEE was performed as there was a strong suspicion that cardiac embolisms were responsible for many of the complications during hospitalization. It showed the presence of a large vegetation, which had both sessile as well as a mobile elongated piece measuring about 2 cm and was attached to the noncoronary cusp of the aortic valve. There was evidence of moderate aortic regurgitation and a moderate pericardial effusion (Figure 5).

On the 16th day of admission, aortic valve replacement with a 23 mm bioprosthesis and drainage of the pericardial effusion were performed by cardiothoracic surgery. Excised aortic valve pathology results showed cardiac valve leaflets with focal, superficial fibrin deposition and detached fibrinous aggregates with polymorphonuclear leukocytes.

On the 23rd day of admission, apixaban 2.5 mg twice a day was ordered as episodes of paroxysmal atrial fibrillation were noted and renal dosing adjustment was needed as our patient had an acute kidney injury. Aspirin was continued. After 13 days in the cardiovascular intensive care unit, the patient was discharged with apixaban, aspirin, colchicine, and prednisone. He was scheduled by oncology for immunotherapy and chemotherapy in four weeks.

**Figure 6:**
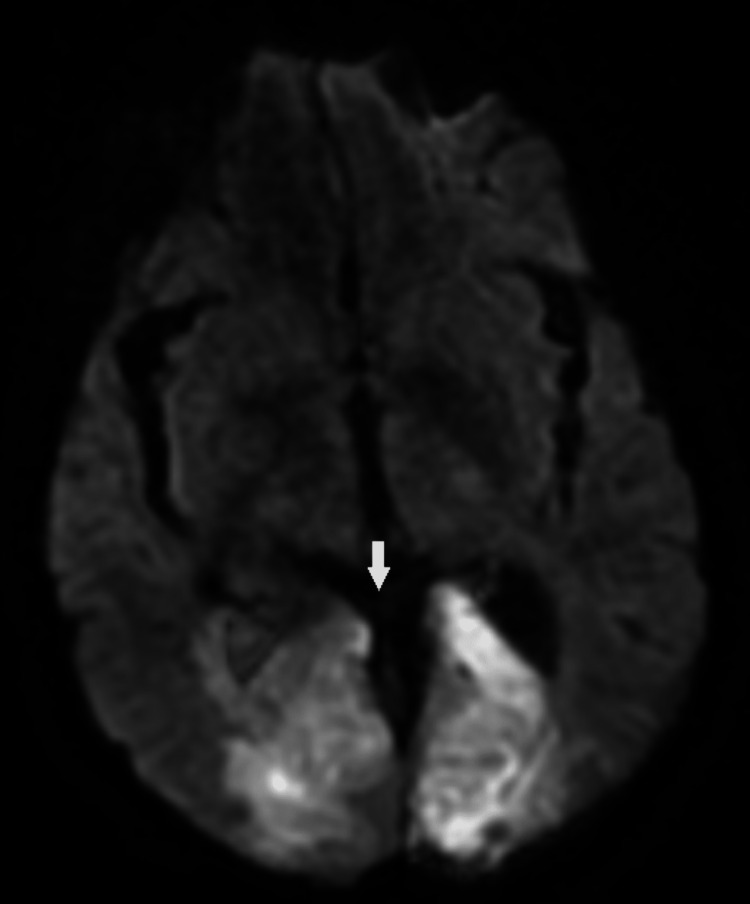
Diffuse-weighted image of the brain showed acute bilateral occipital infarcts (arrow).

He returned four days later with complaints of blurry vision, shortness of breath, and right foot pain. The family reported noncompliance with the discharge medication regimen. Brain MRI reported acute bilateral occipital infarcts (Figure 6). He was found to be in hypoxemic respiratory failure, with respiratory acidosis and hypoglycemia, later becoming unresponsive. The family requested do not resuscitate (DNR)/do not intubate (DNI) for him, and hours later, he expired.

## Discussion

Here, we presented a case of a patient with NBTE who developed widely distributed emboli attributable to 2 cm aortic vegetation in the context of newly diagnosed lung adenocarcinoma. Notably, our patient had undergone a workup for a recent CVA, which included a TEE the month prior that had shown no sign of vegetation. He presented with poorly defined abdominal pain and poor appetite, additionally found to have significant unintentional weight loss. A right lung mass was discovered and subsequently biopsied, showing results consistent with adenocarcinoma.

Noninfectious cardiac valve lesions have been more commonly associated with underlying malignant states, especially with adenocarcinoma and autoimmune diseases like systemic lupus erythematosus and antiphospholipid syndrome [7,8]. Our patient underwent testing for autoimmune etiologies with negative results. Lung biopsy showed morphological and immunohistochemical findings consistent with lung-primary adenocarcinoma, and further testing revealed elevated tumor markers. Direct endothelial injury, thought to be caused by inflammatory cytokines coupled with a hypercoagulable state, is thought to trigger the development of a platelet-fibrin thrombus with a tendency for embolization, as seen in this case with bilateral lower extremity arterial occlusion, acute bilateral occipital lobe infarctions and multiple bilateral renal hypodensities consistent with infarcts [7-9].

NBTE must be distinguished from infective endocarditis even in the absence of systemic signs of infection, especially in the presence of risk factors, as with our patient's reported history of polysubstance abuse. Infective endocarditis was effectively ruled out with negative blood cultures and negative serologic testing for organisms that cause culture-negative endocarditis. During this subsequent hospitalization, an initial transthoracic echocardiogram showed normal systolic function with moderate aortic valve regurgitation and small/moderate pericardial effusion. Transthoracic echocardiogram (TTE) vs. TEE sensitivity can differ, and TTE may fail to detect vegetation in 20-35% of patients, while TEE may detect vegetation in >90% of patients [10]. A TEE was considered but initially delayed since the patient had undergone a TEE with unremarkable results just 40 days prior. Repeat TEE reported an aortic vegetation with a mobile elongated piece measuring about 2 cm, associated with moderate aortic regurgitation. Despite negative study results, strong clinical suspicion should warrant periodic repetition of imaging studies or exploring other imaging modalities, such as electrocardiographic-gated multislice cardiac CTA in combination with TEE, which may be superior to TEE alone [6].

He underwent aortic valve and vegetation excision with bioprosthesis replacement by cardiothoracic surgery. Intraoperative findings described large vegetations in all three leaflets of the aortic valve, with no vegetation seen in any other valve. The final results of surgical cultures (including mediums for mycoses and acid-fast bacilli) of the excised aortic vegetation were negative. Surgical pathology reported focal, superficial fibrin deposition, with detached fibrinous aggregates with polymorphonuclear leukocytes. He also underwent a thrombectomy for his lower extremity arterial occlusion, and systemic anticoagulation was provided with low-dose apixaban and antithrombotic therapy with low-dose aspirin. Treatment consists of systemic anticoagulation and therapy directed at treating the underlying cause. Plans for chemotherapy/immunotherapy in the outpatient setting were made, and our patient was discharged home. Unfortunately, he returned four days later with complaints of blurry vision, shortness of breath, and right foot pain. The family reported noncompliance with the discharge medication regimen. Brain MRI reported acute bilateral occipital infarcts. He was found to be in hypoxemic respiratory failure, with respiratory acidosis, and hypoglycemia, later becoming unresponsive. He was made DNR/DNI by family and expired.

## Conclusions

We present an unusual case of nonbacterial thrombotic endocarditis attributable to malignancy, with a recent negative transesophageal echocardiogram. This was treated with aortic valve replacement and anticoagulation. We highlight the importance of high clinical suspicion in patients with signs of embolization, even when a recent adequate workup has been performed and is unremarkable. Repeat TEE should be considered in these patients. It is essential to diagnose these patients to guide appropriate interventions and prevent complications.
